# The efficacy of oral and subcutaneous antigen-specific immunotherapy in murine cow’s milk- and peanut allergy models

**DOI:** 10.1186/s13601-017-0170-y

**Published:** 2017-09-29

**Authors:** Marlotte M. Vonk, Laura Wagenaar, Raymond H. H. Pieters, Leon M. J. Knippels, Linette E. M. Willemsen, Joost J. Smit, Betty C. A. M. van Esch, Johan Garssen

**Affiliations:** 1 Department of Pharmacology, Faculty of Science, Utrecht Institute for Pharmaceutical Sciences, Utrecht University, Utrecht, The Netherlands; 2 Immunology Platform, Nutricia Research, Utrecht, The Netherlands; 3 Department of Immunotoxicology, Faculty of Veterinary Medicine, Institute for Risk Assessment Sciences, Utrecht University, PO Box 80177, 3508 TD Utrecht, The Netherlands; 4 Yalelaan 104, 3594 CM Utrecht, The Netherlands

**Keywords:** Cow’s milk allergy, Peanut allergy, Immunotherapy, Oral, Subcutaneous, Desensitization, Tolerance induction

## Abstract

**Background:**

Antigen-specific immunotherapy (AIT) is a promising therapeutic approach for both cow’s milk allergy (CMA) and peanut allergy (PNA), but needs optimization in terms of efficacy and safety.

**Aim:**

Compare oral immunotherapy (OIT) and subcutaneous immunotherapy (SCIT) in murine models for CMA and PNA and determine the dose of allergen needed to effectively modify parameters of allergy.

**Methods:**

Female C3H/HeOuJ mice were sensitized intragastrically (i.g.) to whey or peanut extract with cholera toxin. Mice were treated orally (5 times/week) or subcutaneously (3 times/week) for three consecutive weeks. Hereafter, the acute allergic skin response, anaphylactic shock symptoms and body temperature were measured upon intradermal (i.d.) and intraperitoneal (i.p.) challenge, and mast cell degranulation was measured upon i.g. challenge. Allergen-specific IgE, IgG1 and IgG2a were measured in serum at different time points. Single cell suspensions derived from lymph organs were stimulated with allergen to induce cytokine production and T cell phenotypes were assessed using flow cytometry.

**Results:**

Both OIT and SCIT decreased clinically related signs upon challenge in the CMA and PNA model. Interestingly, a rise in allergen-specific IgE was observed during immunotherapy, hereafter, treated mice were protected against the increase in IgE caused by allergen challenge. Allergen-specific IgG1 and IgG2a increased due to both types of AIT. In the CMA model, SCIT and OIT reduced the percentage of activated Th2 cells and increased the percentage of activated Th1 cells in the spleen. OIT increased the percentage of regulatory T cells (Tregs) and activated Th2 cells in the MLN. Th2 cytokines IL-5, IL-13 and IL-10 were reduced after OIT, but not after SCIT. In the PNA model, no differences were observed in percentages of T cell subsets. SCIT induced Th2 cytokines IL-5 and IL-10, whereas OIT had no effect.

**Conclusion:**

We have shown clinical protection against allergic manifestations after OIT and SCIT in a CMA and PNA model. Although similar allergen-specific antibody patterns were observed, differences in T cell and cytokine responses were shown. Whether these findings are related to a different mechanism of AIT in CMA and PNA needs to be elucidated.

## Background

Food allergy is an important socio-economic health problem estimated to occur in 10% of pre-school children (Westernized countries) and 1–2% of adult individuals (USA) [1, 2]. Two of the major allergenic foods, peanut- and cow’s milk protein, show different disease patterns. Cow’s milk allergy (CMA) is most prevalent during early childhood, but is often outgrown [3] while peanut allergy (PNA) is more persistent and is the most frequent cause of life-threatening allergic reactions in adults [4]. Unfortunately, current treatment options for food allergies are limited, being a strict elimination diet and self-administration of epinephrine in case of an anaphylactic response. The need for effective and safe therapeutic options has elicited intensive research into antigen-specific immunotherapy (AIT) as an active tolerance-inducing strategy.

One form of AIT, subcutaneous immunotherapy (SCIT), is effective and safe in respiratory allergies and insect venom hypersensitivities [5–7] and has been recognized as the gold standard immunotherapy method for decades [8]. However, to date, SCIT has not been used to treat food allergies due to the high incidence of severe side effects in two conducted peanut allergy trials [9, 10]. The less invasive alternative, oral immunotherapy (OIT), has been shown to increase the threshold of food tolerated in a double-blind placebo-controlled food challenge (DBPCFC) in a majority of the subjects in several randomized placebo-controlled clinical trials when on therapy [11]. Nonetheless, OIT for food allergy is still an experimental therapeutic strategy because of the risk of side effects and accidental symptoms towards a previously tolerated dose. OIT in cow’s milk- and peanut allergic children was accompanied by persistent adverse reactions during treatment [12, 13]. In addition, sustained unresponsiveness to a food challenge after discontinuation of OIT has only been demonstrated in a minority of the subjects [14]. This clearly leaves OIT open for improvement in both therapy safety and efficacy.

Specific immunological aspects have been suggested to be involved in desensitization and the development of clinical tolerance, including a suppressed T helper 2 (Th2) cell response [11] and the induction of regulatory T cells (Tregs) [15–17], decreased antigen-specific IgE and increased antigen-specific IgG4 levels [18] and effector cell unresponsiveness [19] in mice and/or human. Further attempts to link immunologic changes induced by AIT to clinical protection have been made using murine models of egg allergy. The induction of long-term tolerance was unsuccessful, however, significant changes in intestinal gene expression were observed in clinically protected mice [20, 21]. In humans, clinical tolerance was associated with hypomethylation of the forkhead box protein 3 (FoxP3) locus in Tregs [22].

The goal of this study was to compare the efficacy of OIT and SCIT and to determine the dose of allergen needed to effectively modify parameters of allergy in murine CMA and PNA models.

## Methods

### Mice

All animal procedures were performed according to governmental guidelines and approved by the Ethical Committee of Animal Research of Utrecht University, Utrecht, The Netherlands. Specific-pathogen free 6-week old female C3H/HeOuJ mice (n = 6–8/group) were purchased from Charles River Laboratories (L’Arbresle Cedex, France) and were fed a peanut- and cow’s milk protein-free standard mouse chow (AIN-93G soya, Special Diets Services, Witham, UK). The animals were housed at the animal facility of Utrecht University on a 12 h light/dark cycle with unlimited access to food and water.

### Reagents

Peanut protein extract (PE) was prepared from raw peanuts (provided by Intersnack Nederland BV, The Netherlands) as described previously [23]. Concisely, protein was extracted from ground peanut by mixing 150 g of ground peanut with 750 ml of 20 mM Tris buffer (pH 7.2). After stirring every 10 min for 2 h at room temperature (RT), the aqueous fraction was collected after centrifugation (3000×g for 30 min) and subsequently centrifuged at 10,000×g for 30 min to remove residual traces of fat and insoluble particles. The extract contained 30 mg/ml protein as determined by Bradford analysis with Bovine Serum Albumin (BSA) as a standard. Whey protein powder was provided by Nutricia Research (Utrecht, The Netherlands). Cholera toxin (CT) was purchased from List Biological Laboratories Inc. (Campbell, CA, USA).

### Experimental design: oral sensitization, immunotherapy and challenges

**Fig. 1:**
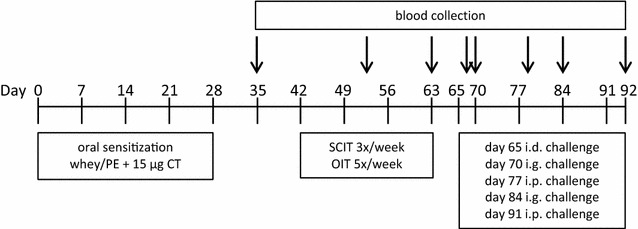
Experimental set-up of PNA and CMA model. Mice were sensitized i.g. to whey, PE or PBS alone in combination with CT (day 0, 7, 14, 21 and 28 for CMA and day 0, 1, 2, 7, 14, 21 and 28 for PNA). From day 42, the mice were treated orally for 5 times/week or subcutaneously for 3 times/week with allergen or PBS alone, for three consecutive weeks (day 42–60). On day 65, all mice were challenged i.d. to determine the acute allergic skin response, anaphylactic shock symptom scores and body temperature levels. On day 70 and 84, i.g. challenges were performed to measure mucosal mast cell degranulation. After i.p. challenges on day 77 and 91, anaphylactic shock symptom scores and body temperature levels were measured. At day 92, the mice were killed with cervical dislocation and blood and organs were collected. *PE* peanut extract, *CT* cholera toxin, *SCIT* subcutaneous immunotherapy, *OIT* oral immunotherapy, *i.d.* intradermal, *i.g.* intragastric, *i.p.* intraperitoneal

Mice were sensitized intragastrically (i.g.) to whey (20 mg in 0.5 ml PBS) or PE (6 mg in 200 µl PBS) using CT (15 μg/mouse) as an adjuvant [23, 24] (day 0, 7, 14, 21 and 28 for whey and day 0, 1, 2, 7, 14, 21 and 28 for PE) (Fig. 1). Sham-sensitized mice received CT in PBS alone. One week after the last sensitization (day 42), the mice were treated orally (OIT: 0.1, 1, 10 and 100 mg whey or 0.15, 1.5 and 15 mg PE in 500 µl PBS) for 5 times/week or subcutaneously (SCIT: 2.5, 10 and 25 μg whey or 1, 10 and 100 µg PE in 200 µl PBS) for 3 times/week, for three consecutive weeks (day 42–60). Sham-sensitized and allergen-sensitized control mice were treated i.g. with PBS alone. On day 65, all mice were challenged intradermally (i.d.) in both ear pinnae with 10 μg whey or 1 µg PE in 20 μl PBS to determine the acute allergic skin response, anaphylactic shock symptom scores and body temperature levels. On day 70 and 84, i.g. challenges (using 50 mg whey or 15 mg PE in 500 µl PBS) were performed to measure mucosal mast cell degranulation in blood samples collected after 30 min. After intraperitoneal (i.p.) challenges on day 77 and 91 (using 100 μg whey or 100 µg PE in 200 µl PBS) anaphylactic shock symptom scores and body temperature levels were measured. At day 92, mice were killed with cervical dislocation and blood and organs were collected.

### Acute allergic skin response, anaphylaxis symptom score and body temperature after challenge

After AIT, on day 65, all mice were anesthetized using inhalation of isoflurane to measure ear thickness in duplicate prior to and 1 h after an i.d. injection with allergen in both ear pinnae. Basal ear thickness (μm) was subtracted from the ear thickness 1 h post-challenge to determine ear swelling as a measure for the acute allergic skin response. Body temperature was measured 30 min after the i.d. challenge using a rectal thermometer and signs of anaphylaxis were scored according to the method described by Li et al. [25]. The anaphylaxis-associated drop in body temperature reaches a maximum at time point 30 min after i.d. challenge. In addition, body temperature was measured every 10 min after the i.p. challenge on day 77, using a rectal thermometer and anaphylaxis was scored at time point 40 min after challenge [25].

### Levels of mMCP-1 and allergen-specific IgE, IgG1, IgG2a in serum

Blood samples were collected at nine specific time points during the animal experiment (day 35, 50, 63, 65, 70, 78, 84, 92) via cheek puncture and after centrifugation (10,000 rpm for 10 min) sera were stored at −20 °C until further analysis.

Levels of whey-specific immunoglobulin (Ig) E, IgG1 and IgG2a were determined by means of ELISA as described previously [24]. Briefly, 96-wells high-binding plates (Costar 3590, Corning Incorporated, Corning, NY, USA) were coated overnight at 4 °C with 100 μl (20 μg/ml) whey in coating buffer (carbonate-bicarbonate buffer, 0.05 M, pH 9.6; Sigma-Aldrich Chemicals, Zwijndrecht, The Netherlands). The plates were washed (PBS with 0.05% Tween20) and blocked for 1 h (RT) in ELISA buffer (50 mM TRIS, 137 mM NaCl, 2 mM EDTA and 0.05% Tween20) with 0.5% BSA. Serum samples were diluted and incubated on the plates for 2 h (RT). After washing, 100 μl biotin-labeled rat anti-mouse IgE, IgG1 and IgG2a (1 μg/ml; BD Biosciences, Alphen aan den Rijn, The Netherlands) was incubated for 1.5 h (RT). Subsequently, plates were washed and incubated with streptavidin poly horseradish peroxidase (Sanquin, Amsterdam, The Netherlands) for 1 h (RT). After washing, a color reaction was initiated by adding o-phenylendiamine (Sigma). The reaction was stopped using 4 M H_2_SO_4_ and optical density was measured with a Benchmark microplate reader (BioRad, Hercules, CA, USA) at 490 nm.

PE-specific IgE, IgG1 and IgG2a levels in serum were detected by ELISA as previously described [23]. Briefly, for IgG1 and IgG2a, 96-wells high-binding plates (Costar 3590, Corning Incorporated, Corning, NY, USA) were coated overnight at 4 °C with 10 µg/ml PE in PBS followed by blocking 1 h (RT) with 0.5% BSA-ELISA buffer. Serum samples were diluted and incubated for 2 h (RT). For detection, AP-coupled anti-IgG1 and anti-IgG2a were added for 1 h (RT). Subsequently, 1 mg/ml p-nitrofenylphosphate in diethanolamine buffer was used for the color reaction, which was stopped with a 10% EDTA solution and absorbance was measured at 405 nm using an Asys expert 96 plate reader (Biochrom, Cambourne, UK).

To measure PE-specific IgE, 96-wells high-binding plates (Costar 3590, Corning Incorporated, Corning, NY, USA) were coated overnight at 4 °C with 1 µg/ml rat anti-mouse IgE (BD Biosciences) followed by blocking for 1 h (RT) with 0.5% BSA-ELISA buffer. Serum samples were diluted and incubated for 2 h (RT). Subsequently, PE-DIG conjugate solution was added for 1 h (RT). The coupling of DIG to PE was performed according to the manufacturer’s instructions. Briefly, the coupled proteins were separated on a Sephadex G-25 column and labeling efficiency was determined by means of spectrophotometry at 280 nm. After incubation for 1 h (RT) with peroxidase-conjugated anti-DIG fragments, a tetramethylbenzidine substrate (0.1 mg/ml) solution was used and the color reaction was stopped with 2 M H_2_SO_4_. Absorbance was measured at 450 nm. Concentrations of IgE, IgG1 and IgG2a were calculated in arbitrary units (AU) using a standard curve of pooled sera from alum-i.p. whey- or PE-sensitized mice.

Mucosal Mast Cell Protease-1 (mMCP-1) was determined by using a mMCP-1 Sandwich ELISA kit (Mouse MCPT-1 Ready-SET-Go!^®^ ELISA, eBioscience, Breda, The Netherlands) according to the manufacturer’s instructions. Levels of mMCP-1 were determined in serum samples obtained 30 min after i.g. challenge.

### Analysis of T cell populations using flow cytometry

After collection and homogenization of the spleen (incl. red blood cell lysis) and the mesenteric lymph nodes (MLN; only in the CMA model), single cell suspensions were used to analyze T cell subsets by flow cytometry. 5–10 × 10^5^ cells per well were collected in fluorescence activated cell sorting (FACS) buffer (PBS containing 0.25% BSA, 0.05% NaN_3_ and 0.5 mM EDTA) and plated. The cells were blocked for 20 min using PBS containing 1% BSA and 5% fetal calf serum (FCS) in the CMA experiment and Fc block (anti-mouse CD16/32 clone 93, eBioscience) in the PNA experiment. Subsequently, cells were stained with the following antibodies in FACS buffer for 30 min at 4 °C: anti-CD4-PerCpCy5.5 (1:100, clone RM4-5), anti-CD25-AlexaFluor 488 (1:100, clone PC61.5), anti-FoxP3-APC (1:50, clone FJK-16s), anti-CD69-APC (1:100, clone H1.2F3), anti-CXCR3-PE (1:50, clone CXCR3-173), anti-CD3e-PerCpCy5.5 (1:100, clone 145-2C11), anti-CD8α-PE (1:100, clone 53-6.7), anti-CD4-FITC (1:200, clone RM4-5), anti-CD25-PE (1:200, clone PC61.5), anti-CD3e-FITC (1:200, clone 145-2C11) from eBioscience, anti-T1/ST2-FITC (1:50, clone DJ8) from mdbioproducts, anti-CD4-FITC (1:100, clone RM4-5), anti-CD4-PerCp (1:200, clone RM4-5), anti-CD8α-PerCp (1:100, clone 53–6.7), anti-CD4-APC (1:200, clone RM4-5), anti-CD69-PE (1:200, clone H1.2F3) from BD Biosciences. Antibody concentrations were individually titrated beforehand and isotype controls were used. Dead and/or aggregated cells were excluded based on forward/sideward scatter properties. Cut-off gates for positivity were established using the fluorescence-minus-one (FMO) technique. Cells stained for extracellular markers were fixed using 1% paraformaldehyde and cells stained for intracellular FoxP3-APC were permeabilized and fixed using the buffer set purchased from eBioscience according to the manufacturer’s protocol. Analysis of the CMA samples was performed on the FACS Canto II (BD Biosciences) and Flowlogic software (Inivai Technologies, Mentone, Australia). Analysis of the PNA samples was performed on the BD Accuri^™^C6 flow cytometer and BD sampler software (BD Biosciences).

### Cytokine release after ex vivo stimulation with whey or PE

8 × 10^5^ cells per well in 200 μl derived from spleen and MLN (in CMA model) were cultured in U-bottom culture plates (Greiner, Frickenhausen, Germany) using RPMI 1640 medium (Lonza, Verviers, Belgium) with 10% FCS, penicillin (100 U/ml)/streptomycin (100 μg/ml) (Sigma) and β-mercaptoethanol (CMA model, 20 μM). All cells received either stimulation with culture medium as a negative control, a polyclonal stimulation with anti-CD3 (CMA model; 1 μg/ml, clone 17A2, eBioscience) or anti-CD3/CD28 (PNA model; 1 μg/ml, clone 145-2C11 and clone 37.51, eBioscience) or antigen-specific stimulation with whey (50 μg/ml) or PE (100 μg/ml). Plates were incubated for 48 h (anti-CD3 or anti-CD3/CD28) or 96 h (whey or PE) to assess production of interleukin (IL)-5, IL-10, IL-13 and Interferon γ (IFNγ) by T cells. Culture supernatants were collected and stored at −20 °C until further analysis with the Ready-SET-Go!^®^ ELISA (eBioscience) according to the manufacturer’s instructions.

### Statistics

The acute allergic skin response, body temperature levels, flow cytometry data, cytokine concentrations and serum mMCP-1 and immunoglobulin levels are depicted as mean ± SEM and were statistically analyzed with GraphPad Prism software version 6.00 (GraphPad software, La Jolla, CA, USA) using one-way ANOVA and Dunnett’s post hoc test for multiple comparisons to compare the treatment groups with the sensitized control animals within each individual experiment. Body temperature curves were statistically analyzed using a repeated measures two-way ANOVA and Dunnett’s post hoc test for multiple comparisons with matched values. Anaphylaxis symptom scores were analyzed using Kruskal–Wallis test for nonparametric data with Dunn’s post hoc test. Results were considered statistically significant when P < 0.05.

## Results

### Reduction in allergic manifestations upon challenge with whey or PE in OIT and SCIT mice

**Fig. 2:**
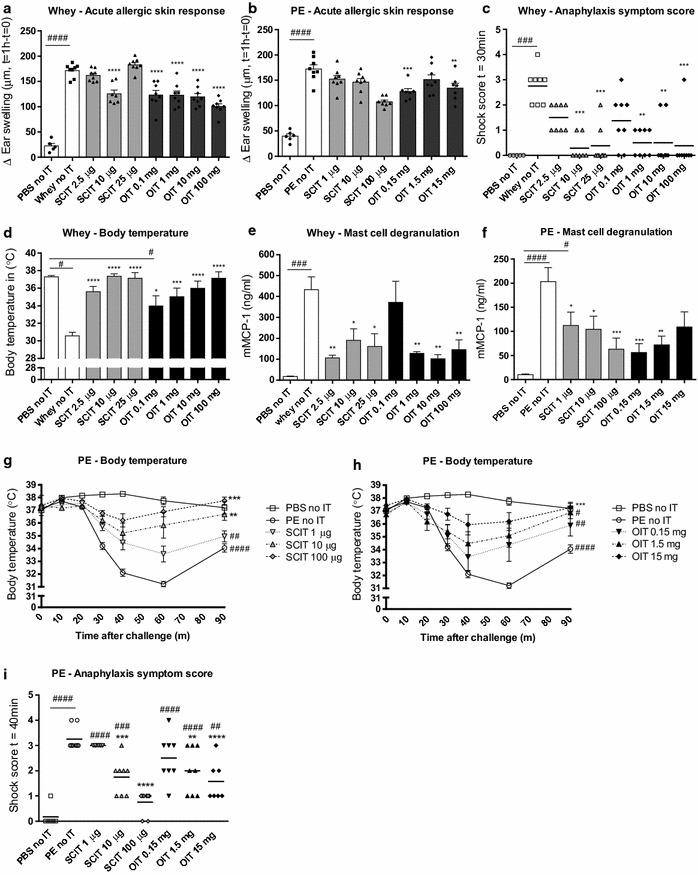
Allergic manifestations evaluated in whey- or PE-sensitized mice after receiving SCIT and OIT. **a**, **b** Acute allergic skin response measured as Δ ear swelling 1 h after i.d. challenge. **c** Anaphylactic shock symptom scores determined 30 min after i.d. challenge in CMA model. **d** Body temperature measured 30 min after i.d. challenge in CMA model. **e**, **f** Concentrations of mMCP-1 in serum collected 30 min after i.g. challenge. **g**, **h** Change in body temperature after i.p. challenge in PNA model. **i** Anaphylactic shock symptom scores determined 40 min after i.p. challenge in PNA model. Data are represented as mean ± SEM n = 6–8 mice/group. Statistical analysis was performed using one-way ANOVA and Dunnett’s post hoc test for multiple comparisons or a repeated measures two-way ANOVA and Dunnett’s post hoc test for multiple comparisons with matched values for the temperature curve in g–h. ^#^P < 0.05; ^##^P < 0.01; ^###^P < 0.001; ^####^P < 0.0001 compared to sham control. *P < 0.05; **P < 0.01; ***P < 0.001; ****P < 0.0001 compared to whey- or PE-sensitized control. *OIT* oral immunotherapy, *SCIT* subcutaneous immunotherapy, *PE* peanut extract; *CT* cholera toxin, *mMCP-1* mucosal mast cell protease-1, *IT* immunotherapy

The acute allergic skin response, measured as ear swelling after i.d. injection with whey or PE, was increased in sensitized mice (whey/PE no IT) compared to sham-sensitized mice (PBS no IT) (Fig. 2a, b). In the CMA model, SCIT reduced the acute allergic skin response only at a dose of 10 μg, whereas OIT reduced acute allergic skin responses at all dosages (Fig. 2a). Furthermore, both SCIT (10, 25 μg) and OIT (1, 10, 100 mg) reduced anaphylactic shock symptom scores (Fig. 2c). SCIT and OIT prevented the characteristic drop in body temperature observed during anaphylaxis and this effect appeared to be dose-related (Fig. 2d). In the PNA model, SCIT (100 μg) and OIT (0.15, 15 mg) reduced the acute allergic skin response (Fig. 2b). No anaphylactic response was induced after i.d. administration of the used PE dosage, therefore no body temperature and anaphylactic shock symptom score data were included. The i.p. challenge performed in PE-sensitized mice on day 77 indicated protection against clinical responses in a dose-related manner after both SCIT and OIT. Both the drop in body temperature (Fig. 2g, h) and the anaphylactic shock symptom scores (Fig. 2i) were significantly reduced in all AIT groups. The i.p. challenge performed in the whey-sensitized mice on day 77 did not show protection against clinical signs, since the used dose of 100 μg induced severe anaphylaxis in all groups (data not shown). In addition, a second i.p. challenge performed on day 91 in both food allergy models did not induce an anaphylactic response in allergen-sensitized control animals (data not shown).

To determine the effect of OIT and SCIT on the local effector response in the gastrointestinal tract, mMCP-1 concentrations were measured in serum collected 30 min after i.g. challenge (day 70) (Fig. 2e, f). Mast cell degranulation was reduced in all treatment groups in the CMA model, except OIT with 0.1 mg whey (Fig. 2e). A second i.g. challenge (day 84) did not induce detectable mMCP-1 levels in serum (data not shown). In PE-sensitized animals, a reduction in mMCP-1 was observed in all SCIT groups and the 0.15 and 1.5 mg OIT groups (Fig. 2f). The second i.g. challenge (day 84) showed a similar induction of mMCP-1 in sensitized animals and this increase was absent in SCIT (100 μg) and OIT (0.15, 15 mg, data not shown). In short, SCIT and OIT induced clinical protection against food challenges in both the CMA and PNA model.

### Induction of allergen-specific IgE upon challenge absent in OIT and SCIT mice

**Fig. 3:**
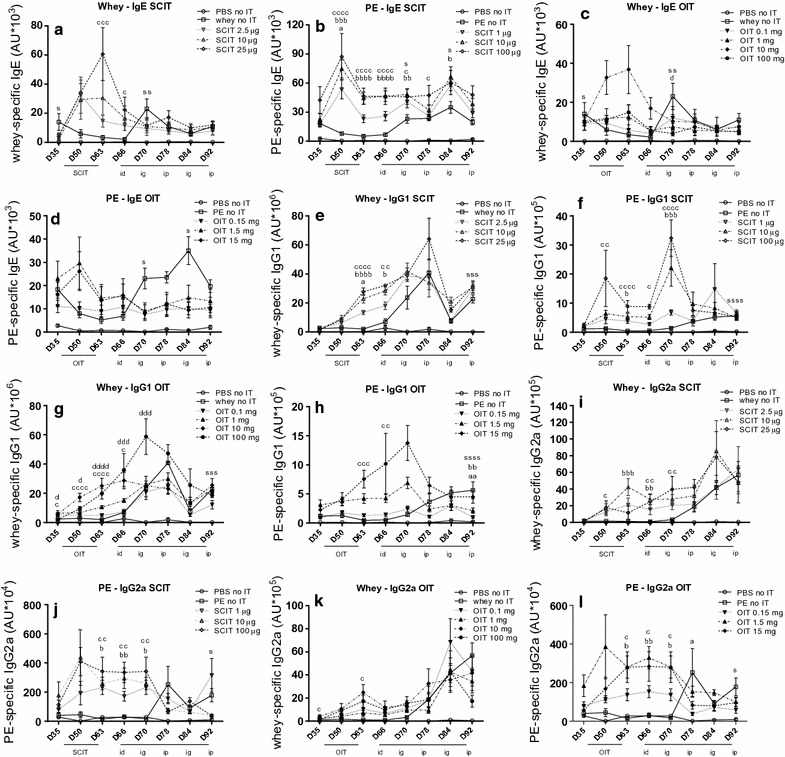
Allergen-specific IgE, IgG1 and IgG2a levels in serum determined by ELISA. **a**, **b** Allergen-specific IgE in SCIT groups. **c**, **d** Allergen-specific IgE in OIT groups. **e**, **f** Allergen-specific IgG1 in SCIT groups. **g**, **h** Allergen-specific IgG1 in OIT groups. **i**, **j** Allergen-specific IgG2a in SCIT groups. **k**, **l** Allergen-specific IgG2a in OIT groups. Data are represented as mean ± SEM n = 6–8 mice/group. Statistical analysis was performed on each individual time point using one-way ANOVA and Dunnett’s post hoc test for multiple comparisons. All treatment groups were compared to the sensitized control group and significant differences were indicated with letters e.g. ^a^P < 0.05; ^aa^P < 0.01; ^aaa^P < 0.001; ^aaaa^P < 0.0001. In CMA figures: a for SCIT 2.5 μg and OIT 0.1 mg, b for SCIT 10 μg and OIT 1 mg, c for SCIT 25 μg and OIT 10 mg, d for OIT 100 mg and s for sham control. In PNA figures: a for SCIT 1 μg and OIT 0.15 mg, b for SCIT 10 μg and OIT 1.5 mg and c for SCIT 100 μg and OIT 15 mg and s for sham control. *OIT* oral immunotherapy, *SCIT* subcutaneous immunotherapy, *IT* immunotherapy, *PE* peanut extract, *id* intradermal challenge, *ig* intragastric challenge, *ip* intraperitoneal challenge

Allergen-specific IgE, IgG1 and IgG2a levels in serum were measured in particular to investigate whether OIT and SCIT modulated the humoral response. During and after AIT (day 50, 63), SCIT increased allergen-specific IgE levels in the PNA model (Fig. 3b) and in the CMA model with a dose of 25 μg (Fig. 3a). At day 70, 5 days after the i.d. challenge, the rise in allergen-specific IgE observed in sensitized control animals compared to sham-sensitized control animals was absent in the OIT and SCIT mice (Fig. 3a–d). In addition, OIT with 100 mg whey showed significantly lower whey-specific IgE levels compared to the whey-sensitized control group (Fig. 3c). High dose SCIT and OIT induced IgG1 and IgG2a in the CMA and PNA model (day 63, Fig. 3e–l). The induction of allergen-specific IgG1 and IgG2a was delayed in sensitized control mice; an increase was observed after the i.d. challenge (day 70) and levels appeared to continuously rise upon repeated challenges (day 70, 78, 84 and 92). However, allergen-specific IgG1 levels in the SCIT and OIT mice did not further increase after day 70/78 (Fig. 3e–h) despite the challenges. In summary, the data demonstrate that for both allergens OIT and SCIT protected against a challenge-induced rise in allergen-specific IgE and induced allergen-specific IgG1 and IgG2a during immunotherapy.

### Shifted T cell profile in lymph organs after OIT and SCIT

**Fig. 4:**
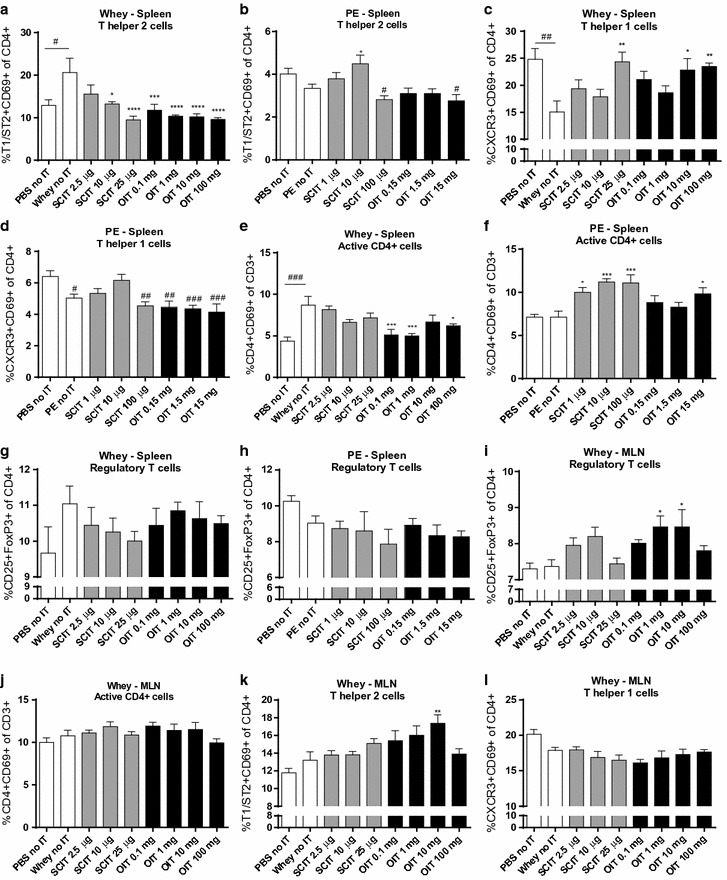
Flow cytometric analysis of T cell populations in the spleen and MLN. Cells were gated based on FSC–SSC properties and the Fluorescence-minus-one (FMO) technique. **a**, **b** Percentage of activated Th2 cells (T1/ST2+ CD69+ of CD4+) in spleen. **c**, **d** Percentage of activated Th1 cells (CXCR3+ CD69+ of CD4+) in spleen. **e**, **f** Percentage of activated CD4+ cells (CD4+ CD69+ of CD3+) in spleen. **g**, **h** Percentage of Tregs (CD25+ FoxP3+ of CD4+) in spleen. **i** Percentage of Tregs (CD25+ FoxP3+ of CD4+) in MLN of CMA animals. **j** Percentage of activated CD4+ cells (CD4+ CD69+ of CD3+) in MLN of CMA animals. **k** Percentage of activated Th2 cells (T1/ST2+ CD69+ of CD4+) in MLN of CMA animals. **l** Percentage of activated Th1 cells (CXCR3+ CD69+ of CD4+) in MLN of CMA animals. All data are represented as mean ± SEM n = 6/8 mice/group. Statistical analysis was performed using one-way ANOVA and Dunnett’s post hoc test for multiple comparisons. ^#^P < 0.05; ^##^P < 0.01; ^###^P < 0.001; ^####^P < 0.0001 compared to sham control control. *P < 0.05; **P < 0.01; ***P < 0.001; ****P < 0.0001 compared to whey- or PE-sensitized control. PE, peanut extract; OIT, oral immunotherapy; SCIT, subcutaneous immunotherapy; IT, immunotherapy

In the spleen, the percentage of activated Th2 cells (T1/ST2+ CD69+ of CD4+ cells) was elevated in whey-sensitized control animals compared to sham-sensitized control animals (Fig. 4a). OIT (all dosages) and SCIT (10, 25 μg) reduced the percentage of activated Th2 cells. This reduction coincided with an increase in the percentage of activated Th1 cells (CXCR3+ CD69+ of CD4+ cells) in the 25 μg SCIT and 10 mg and 100 mg OIT groups (Fig. 4c) compared to the whey-sensitized control animals. In contrast to the CMA model, no difference in the percentage of activated Th2 cells was observed in PE-sensitized control animals compared to sham-sensitized control animals (Fig. 4b). The percentage of activated Th1 cells was decreased in PE-sensitized mice compared to sham-sensitized mice (Fig. 4d). In addition, SCIT and OIT with PE (15 mg) increased the percentage of activated CD4+ T cells (CD4+ CD69+ of CD3+ cells) compared to the PE-sensitized control animals (Fig. 4f).

In the MLN collected in the CMA model, the percentage of CD4+ CD25+ FoxP3+ Tregs was elevated in OIT mice (1, 10 mg) (Fig. 4i) compared to whey-sensitized control animals. In addition, an increase in percentage of activated Th2 cells in the 10 mg OIT group was observed. No effect of OIT and SCIT on the induction of Tregs was found in spleen in either the CMA or the PNA model (Fig. 4g, h). Briefly, we observed differences in the percentages of T cells in the lymph organs of the cow’s milk and peanut allergic mice in response to therapy.

### Altered cytokine production after ex vivo stimulation of lymphocytes with whey or PE

**Fig. 5:**
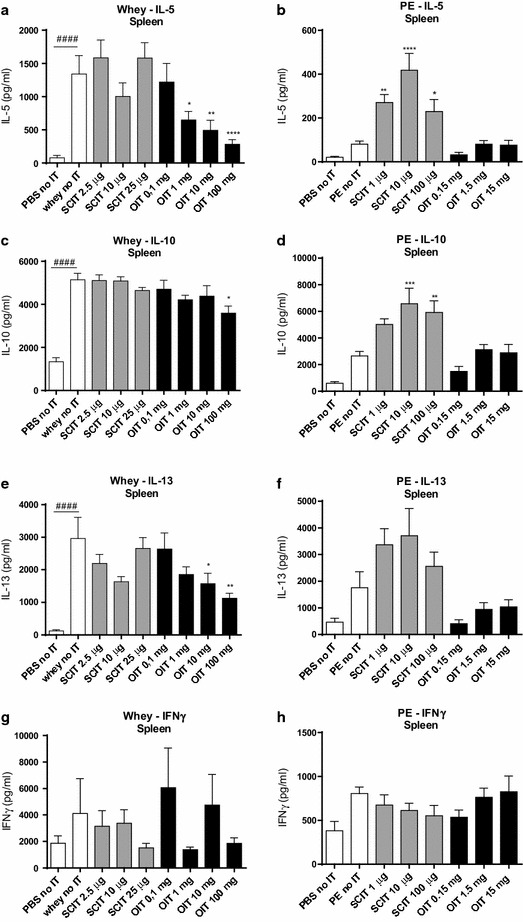
Cytokine concentrations after ex vivo stimulation of splenocytes with whey or PE determined by ELISA. Splenocytes were cultured for 96 h in the presence of PE, whey or medium (medium data not shown). **a**, **b** IL-5 concentration. **c**, **d** IL-10 concentration. **e**, **f** IL-13 concentration. **g**, **h** IFNγ concentration. Data are represented as mean ± SEM n = 6/8 mice/group. Statistical analysis was performed using one-way ANOVA and Dunnett’s post hoc test for multiple comparisons. ^#^P < 0.05; ^##^P < 0.01; ^###^P < 0.001; ^####^P < 0.0001 compared to sham-sensitized control. *P < 0.05; **P < 0.01; ***P < 0.001; ****P < 0.0001 compared to whey- or PE-sensitized control. *PE* peanut extract, *OIT* oral immunotherapy, *SCIT* subcutaneous immunotherapy, *IT* immunotherapy

Cellular activation was confirmed in the control conditions of the ex vivo stimulation assay; a significant increase in IL-5, IL-10, IL-13 and IFNγ production was observed in all groups after polyclonal stimulation of cultured cells using anti-CD3 (CMA model) or anti-CD3/CD28 (PNA model) compared to stimulation with only medium in both food allergy models (data not shown). *Ex vivo* stimulation with whey increased the IL-5, IL-10 and IL-13 concentration in supernatants of splenocyte cultures derived from whey-sensitized control animals (Fig. 5a, c, e). OIT (1, 10 and 100 mg) reduced the release of IL-5 and IL-13. Except for 100 mg OIT, IL-10 levels remained high in the OIT groups. SCIT did not change cytokine levels. The Th1-related IFNγ release upon stimulation did not differ among the groups (Fig. 5g). In the PNA model, cytokine production was affected by SCIT but not by OIT (Fig. 5b, d, f, h). Compared to PE-sensitized control mice, SCIT increased antigen-induced release of IL-5 at 1 and 10 µg (Fig. 5b) and IL-10 at 10 and 100 µg (Fig. 5d). No change in IL-13 production was observed in the SCIT groups (Fig. 5f). Again, IFNγ release upon stimulation did not differ between groups (Fig. 5h). Overall, SCIT induced an increase (IL-5, IL-10) in the PNA model, whereas SCIT in the CMA model did not affect cytokine levels. OIT did not affect cytokine production in the PNA model, whereas it reduced Th2-associated cytokine production in the CMA model.

## Discussion

We aimed to compare the efficacy of OIT and SCIT in models of PNA and CMA and to determine the dose of allergen needed to effectively modify parameters of allergy. We have shown in both models that OIT and SCIT reduced clinical manifestations of food allergy and resulted in comparable changes in serum levels of allergen-specific IgE and IgG subtypes. Differences in T cell populations and cytokine profiles suggest a potential difference in the mechanism of AIT for PNA and CMA.

While both types of immunotherapy were able to decrease allergic manifestations upon challenge, the effective therapeutic dose differed per allergen. OIT using 0.1 mg whey did not decrease signs of anaphylaxis and only mildly prevented the drop in body temperature. The efficacy of OIT was evident in the groups receiving 1 mg whey or higher. In SCIT, 10 μg whey was the most effective dose. In the PNA model, the intermediate and high dosages (1.5, 15 mg OIT and 10, 100 μg SCIT) were the most effective in modulation of disease parameters.

To investigate the effect of OIT and SCIT on mucosal mast cell degranulation, all mice were challenged per oral gavage. Although OIT with 0.1 mg whey was ineffective, SCIT and OIT effectively reduced mMCP-1 levels in both food allergy models. This finding indicates that regular administration of an allergen dose above a certain threshold influences responsiveness of effector cells along the gastrointestinal tract. This is a known effect of AIT and reflects desensitization. Repeated stimulation of the FcεRI present on basophils during OIT in peanut allergic individuals reduces basophil activation as shown by down-regulation of the activation marker CD63 [26]. Another possible explanation for the reduced release of mediators by effector cells might be a reduced number of basophils and mast cells in the early phase of immunotherapy [19]. In addition, repeated exposure to the allergen can contribute to exhaustion of effector cells [27]. In the CMA model, a second i.g. challenge after 2 weeks resulted in a low mMCP-1 concentration in the serum of the whey-sensitized control group. This observation might be associated with the low levels of allergen-specific IgE in serum of the whey-sensitized control animals on day 84, while whey-specific IgG2a levels were increased. Such low IgE levels may be insufficient to re-sensitize mucosal mast cells [28]. In vitro studies showed that mast cells are able to refill their granules and respond again to an allergen challenge after 24–48 h [29]. In accordance, PE-specific IgE levels in serum of the sensitized control animals were not decreased and IgG2a levels were not elevated at day 84 and similar mMCP-1 levels were found upon a second i.g. challenge when compared to the first i.g. challenge. Although protection against i.p. challenge-induced anaphylaxis was shown in OIT and SCIT mice in the PNA model (day 77), a second i.p. challenge did not induce anaphylaxis in allergen-sensitized animals (both CMA and PNA model, day 91). We hypothesized that repeated systemic (i.p.) challenges, as conducted in the current models to ensure a detectable Th2 cell-mediated effector response, leads to exhaustion of the effector cells present in the peritoneum and this unintentionally affects allergic outcomes.

In humans, AIT is known to increase antigen-specific IgE in serum. However, if the treatment is prolonged for a period of months or even years, IgE levels tend to decline [30]. Peanut allergic individuals subjected to OIT showed an initial increase in IgE, but levels were stabilized despite an oral food challenge [31]. In accordance, our findings indicate an increase in allergen-specific IgE in mice receiving OIT and SCIT, followed by a return to baseline when immunotherapy was discontinued. Remarkably, IgE levels failed to increase after the allergen challenges in the SCIT and OIT groups. The induction of allergen-specific IgG1 and IgG2a by OIT and SCIT appeared to be dose-dependent. In humans, IgG4 levels are elevated during immunotherapy and are associated with protection against clinical symptoms [31, 32]. IgG subtypes are proposed to capture the antigen and thereby inhibit binding to IgE present on mast cells and basophils and thus prevent degranulation [19]. The reduced mMCP-1 release measured after i.g. challenge might be explained by the elevated IgG subtype levels in serum. Furthermore, IgG levels were increased by the challenge protocol, including in the serum of the allergen-sensitized control animals. This finding might explain the absence of clinical signs in the follow-up challenges in the CMA model.

The effect of OIT and SCIT on the humoral response can be linked to the percentages of T helper cell subsets in the lymphoid organs in the CMA model, but not in the PNA model. Specific IgE production by plasma cells is sustained by a Th2 cell dominated immune response in the presence of IL-4, IL-5 and IL-13 [33]. Clinical protection after OIT in peanut allergic subjects in a randomized controlled study was accompanied by a reduction in IL-5 and IL-13 concentrations [31]. Skewing of the immune response from a Th2 profile towards a more regulatory profile is associated with a modified cytokine milieu [34]. The reduced percentage of activated Th2 cells in the spleen of OIT mice (CMA model) was accompanied by a dose-dependent reduction in the IL-5 and IL-13 concentration in stimulated cultures. This observation is consistent with the fact that exposure to a high allergen dose leads to anergy in specific T-cells [35]. On the contrary, IL-5 and IL-13 production was not decreased in the SCIT groups, although the percentage of activated Th2 cells was decreased in the 10 and 25 μg groups. These findings suggest that the route of antigen administration is important in the modulation of specific T cell responsiveness during immunotherapy. Th2-associated cytokine IL-4 was not detected in the stimulated cell cultures, as was previously described for the current food allergy model with the C3H/HeOuJ strain [36]. A similar pattern of IL-5 and IL-13 levels as described for spleen was observed in MLN culture supernatants in the CMA model, indicating a suppressed Th2 responsiveness after OIT but not after SCIT. Nevertheless, a tendency towards an increase in activated Th2 cells was observed in the MLN of the OIT groups, with a significant difference in the OIT 10 mg group. In the PNA model, SCIT increased levels of IL-5 and IL-10 compared to the PE-sensitized control animals, whereas OIT did not have an effect on cytokine production. Earlier studies show that tolerance induction by SCIT is accompanied by a shift from a Th2 cytokine profile towards a Th1 cytokine profile, but there are discrepancies in the literature [37]. The observed differences in Th2 cytokine production between OIT and SCIT and between the CMA and PNA models might be explained by the induction of antigen-specific Tregs that can exert a suppressive function towards effector T cells [38, 39].

High dose SCIT and OIT induced a shift in the percentages of activated splenic Th1 and Th2 cells after the final i.p. challenge in the CMA model but not in the PNA model. The observation in the CMA model is in accordance with the hypothesis that oral tolerance induction is characterized by a shift from a Th2 response towards a Th1 response [33]. Furthermore, low-dose induction of tolerance is accompanied by increased numbers of CD4+ CD25+ FoxP3+ Tregs [33]. Indeed, this was observed in the CMA model where OIT (1, 10 mg) increased the percentage of CD4+ CD25+ FoxP3+ Tregs in the MLN. The fact that no difference in percentage of FoxP3+ Tregs was observed in spleen (PNA and CMA), is contradictory to the results published by Dioszeghy et al. [17], who have shown an increase in FoxP3+ Tregs in the spleen of peanut-allergic mice subjected to OIT. This dissimilarity might be explained by the different mouse strains used in both studies; C3H/HeOuJ and BALB/c show differences in allergic responses [36]. In addition, we could not link splenic ex vivo IL-10 levels to the presence of Tregs in both food allergy models. An increase in the IL-10 concentration was found in allergen-stimulated cultures derived from lymph organs of allergic mice, indicating the contribution of Th2-derived IL-10 [40, 41]. Hence, clinical protection observed after AIT and allergen challenge in both the CMA and PNA models might partially be explained by the induced IgG1 and IgG2a levels in combination with low IgE levels. Given the fact that IgG can also drive an alternative food-induced anaphylaxis pathway [42], the potential protective effect of IgG1 and IgG2a needs to be confirmed with a more mechanistic approach.

Despite differences between the CMA and PNA models, overall, the reported clinical, cellular and humoral data can be linked to our current understanding of oral tolerance and immunotherapy mechanisms in humans [19]. However, future use of both models would require further investigation of the exact role of immunoregulatory mechanisms, such as regulatory T cells or antibody-mediated protection, and of long term effects of the therapeutic strategies.

## Conclusion

In conclusion, the murine CMA and PNA studies showed that clinical protection can be achieved via OIT and SCIT. Although similar allergen-specific immunoglobulin patterns were observed, differences in T cell populations and cytokine responses were shown. More insight into the mechanism of (long term) tolerance induction is needed, nonetheless, our findings contribute to the development of effective AIT protocols. In the future, the current OIT models will be used to study the possible benefit of using immunomodulatory food components (e.g. non-digestible oligosaccharides) as adjunct therapy to support antigen-specific immunotherapy in terms of efficacy and safety.

## Abbreviations

AIT: antigen-specific immunotherapy
CMA: cow’s milk allergy
PNA: peanut allergy
OIT: oral immunotherapy
SCIT: subcutaneous immunotherapy
FoxP3: forkhead box protein 3
i.g.: intragastric
PE: peanut extract
CT: cholera toxin
i.d.: intradermal
i.p.: intraperitoneal
Ig: immunoglobulin
Th: T helper
IL: interleukin
DBPCFC: double-blind placebo-controlled food challenge
Treg: regulatory T cell
mMCP-1: murine mast cell protease-1
IFNγ: Interferon γ
MLN: mesenteric lymph nodes
IT: immunotherapy
